# Author Correction: Spatially-optimized urban greening for reduction of population exposure to land surface temperature extremes

**DOI:** 10.1038/s41467-023-41639-2

**Published:** 2023-09-18

**Authors:** Emanuele Massaro, Rossano Schifanella, Matteo Piccardo, Luca Caporaso, Hannes Taubenböck, Alessandro Cescatti, Gregory Duveiller

**Affiliations:** 1 European Commission, Joint Research Centre (JRC), Ispra, Italy; 2 University of Turin, Turin, Italy; 3 ISI Foundation, Turin, Italy; 4 Collaborator of the European Commission, Joint Research Centre (JRC), Ispra, Italy; 5 National Research Council of Italy, Institute of BioEconomy (CNR-IBE), Rome, Italy; 6 German Aerospace Center (DLR), Munich, Germany; 7 University of Würzburg, Würzburg, Germany; 8 Max Planck Institute for Biogeochemistry, Jena, Germany

**Keywords:** Climate-change mitigation, Climate sciences, Mathematics and computing

Correction to: *Nature Communications* 10.1038/s41467-023-38596-1, published online 22 May 2023

The original version of this Article contained an error in Fig. 2, in which the wrong acronym (“HSLN”) was used for the variable of population exposure. The wrong acronym has now been completely removed from Fig. 2A, and it has been replaced with the correct acronym (“TE”) in the legend of Fig. 2B. This has been corrected in both the PDF and HTML versions of the Article.

