# Supplementary material for: Spatially-optimized urban greening for reduction of population exposure to land surface temperature extremes
Source: Nat Commun. 2023 May 22;14:2903. doi: 10.1038/s41467-023-38596-1 (PMC10203342; doi:10.1038/s41467-023-38596-1)
Supplement: Supplementary file 1 — Supplementary Information [file 41467_2023_38596_MOESM1_ESM.pdf]

Supplementary Information for
Spatially-optimized urban greening for reduction of population exposure to land surface temperature extremes

Emanuele Massaro[1,*], Rossano Schifanella[2,3], Matteo Piccardo[4], Luca Caporaso[1,5], Hannes Taubenböck[6,7], Alessandro Cescatti[1], and Gregory Duveiller[1,8]

[1]European Commission, Joint Research Centre (JRC), Ispra, Italy
[2]University of Turin, Turin, Italy
[3]ISI Foundation, Turin, Italy
[4]Collaborator of the European Commission, Joint Research Centre (JRC), Ispra, Italy
[5]National Research Council of Italy, Institute of BioEconomy (CNR-IBE), Rome, Italy
[6]German Aerospace Center (DLR), Munich, Germany
[7]University of Würzburg, Würzburg, Germany
[8]Max Planck Institute for Biogeochemistry, Jena, Germany
[*]emanuele.massaro@ec.europa.eu

Table of contents

This document contains supporting information material to the main text.

This supplementary file includes

Supplementary Notes
Supplementary Figures S1-S10
Supplementary Tables S1-S2
Supplementary References

Supplementary Notes

Climate classification

From the orginal Köppen climate classification [1] we define five main clusters as shown in Figure S1:

- **Arid**: this type of climate is defined by little precipitation and it contains the 7 sub zones such as BWh, BWk, BSh, BSk, Csa, Csb, Csc.
- **Continental**: this type of climate has at least one month on average below $0°C$ and at least one month on average above $10°C$. It contains all the D subclasses.
- **Polar**: In this type of climate, the average temperature is below $10°C$ in all months of the year and is defined by subzones E. In our analysis, we do not observe cities in this climate cluster.
- **Temperate**: For this climate type, the coldest month averages between $0°C$ and $18°C$ and at least one month averages above $10°C$. It grouped the following sub zones: BWh, BWk, BSh, BSk, Csa, Csb, Csc.
- **Tropical**: This type of climate has an average temperature of $18°C$ or higher in each month of the year, with significant precipitation and it is described by the A sub zones.

The spatial lag model

For an individual observation, the spatial lagged equation (written in matrix notation in Eq. **??**) can be written as the following:

$$y_i = \sum_{q=1}^{Q} \beta_q x_{iq} + \rho \sum_{j=1}^{n} w_{ij} y_j + \varepsilon_i \tag{S1}$$

with $j \neq i$, Q defines the number of independent parameters and n corresponds to the number of observed neighbors defined by the approach: in this research we used a k-neighbors approach with $k = 8$, thus $n = k = 8$. Since the dependent variables, y_i, appear on both sides of the expression:

$$Y = X\beta + \rho WY + \varepsilon \tag{S2}$$

we can re-arrange this expression to solve for Y:

$$Y = (I - \rho W)^{-1} X\beta + (I - \rho W)^{-1} \varepsilon \tag{S3}$$

The design of this kind of mixed model incorporates spatial autocorrelation together with the influence of other (aspatial) predictor variables. The objective of this revised approach is to obtain a significant improvement over a standard OLS model. The level of improvement will depend on how well the revised model represents or explains the source data, and to an extent this will vary depending on the detailed form of the weighting matrix, W. The spatial weight matrix W is key to any spatial regression. It defines the relationships between the units of analysis so that the model can control for the fact that areas that are near to each other are more similar than areas that are far apart [2]. Rook adjacency neighbors are defined as those that share a common edge, and queen adjacency neighbors are defined as those that share a common edge or node. The number of links created in a queen matrix will always be at least as large as the number from a rook matrix. We used the Python Pysal library for the spatial analysis [3].

Diagnostics for spatial dependence

We assessed the spatial autocorrelation of the variables using six different tests comprises of Moran's I (error), lagrange multiplier (lag), robust LM (lag), lagrange multiplier (error), robust LM (error), and lagrange multiplier (SARMA) [4]. We did that using Pysal [3]. At p-value of 0.001, the Moran's I score of 0.93 is highly significant, indicating strong spatial autocorrelation of the residuals. The robust LM statistics for spatial lag and spatial error dependence are found to be significant, clearly indicating presence of spatial autocorrelation (Table S1).

Cross-validation setting

In order to validate the models we use a k-fold cross validation scheme that consists in three main phases: i) training, ii) validation and iii) and test. As shown in Figure S7 we firstly define 5 sub-datasets containg the 20% of cities (10 cities for each climate zone). The cities of the Test 1 do not appear in the other subsets and viceversa. For each of the test sets we define a training/validation subset that contains all the cities except the ones in the selected test set. For each phase we then consider a training/validation dataset of 160 cities (40 cities for each climate zone). In this dataset we perform a k-fold cross validation

with $k = 5$ and we evaluate the performance of the models in the training and validation subsets. We choose the values of the models' parameters that optimize the value of the R^2 in the validation setting and we test the models using the relative Test subsets. In the Figure S7 we show the case for the Test 1. We repeat the same analysis 5 times for all the Test subsets and we finally have 25 values of the R^2 for the training and validation steps and 5 values for the test sets. We then use the average values of the parameters β to perform the mitigation strategy analysis.

Supplementary Figures

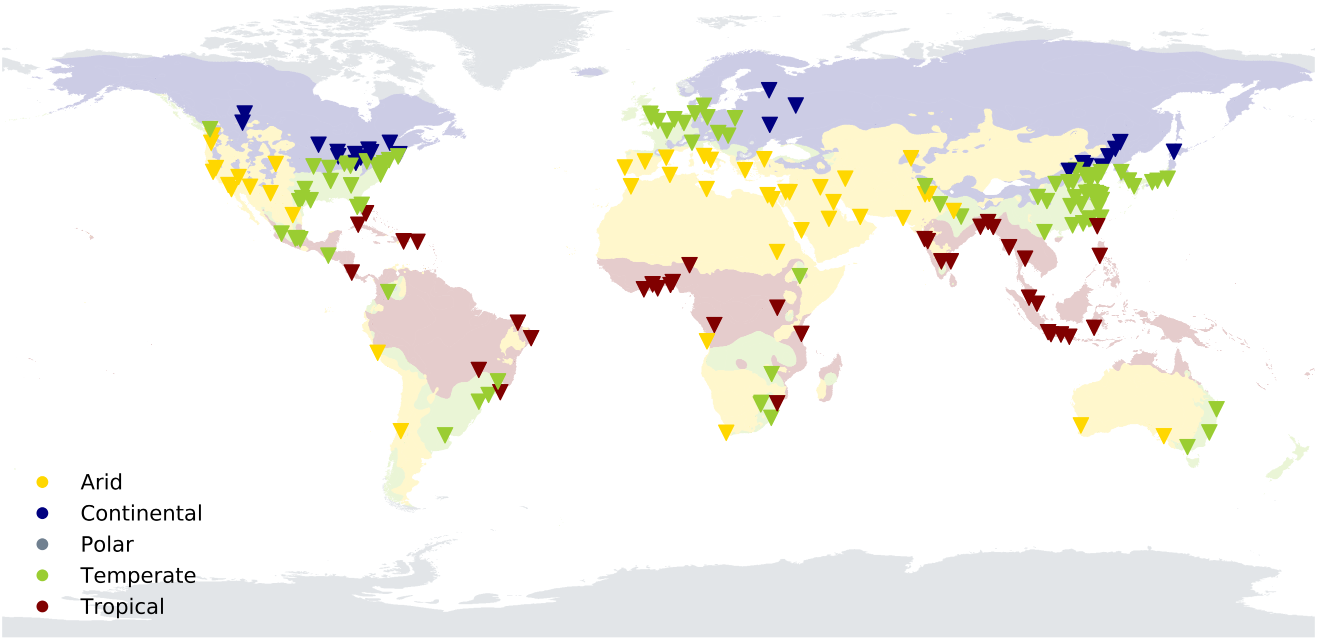

Figure S1. Cities grouped in the climate clusters defined from the Köppen climate classification.

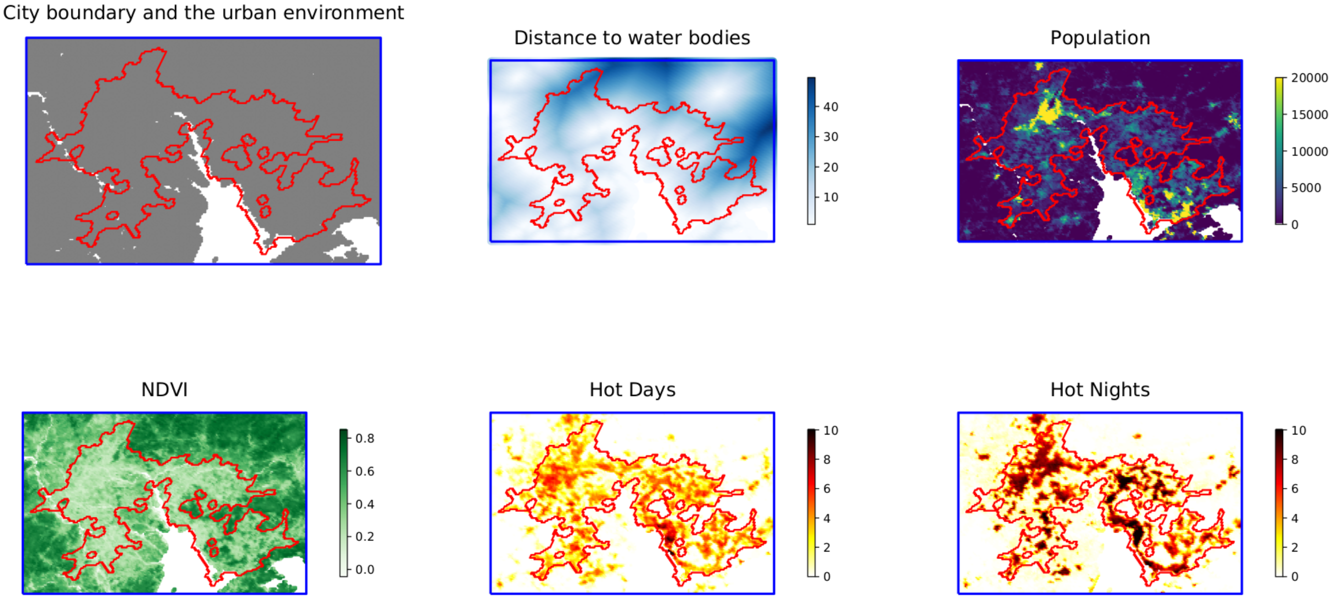

Figure S2. Data used for the regression model for the city of Guangzhou. From the top left to the bottom right: the definition of urban boundary starting from the definition of cities by the Global Human Settlment Layer (GSHL) [5] in red, the urban boundary in gray, heatmaps of the distance to water bodies in kilometers (d_w), population, Normalized Difference Vegetation Index (NDVI) and number of days and nights over threshold respectively.

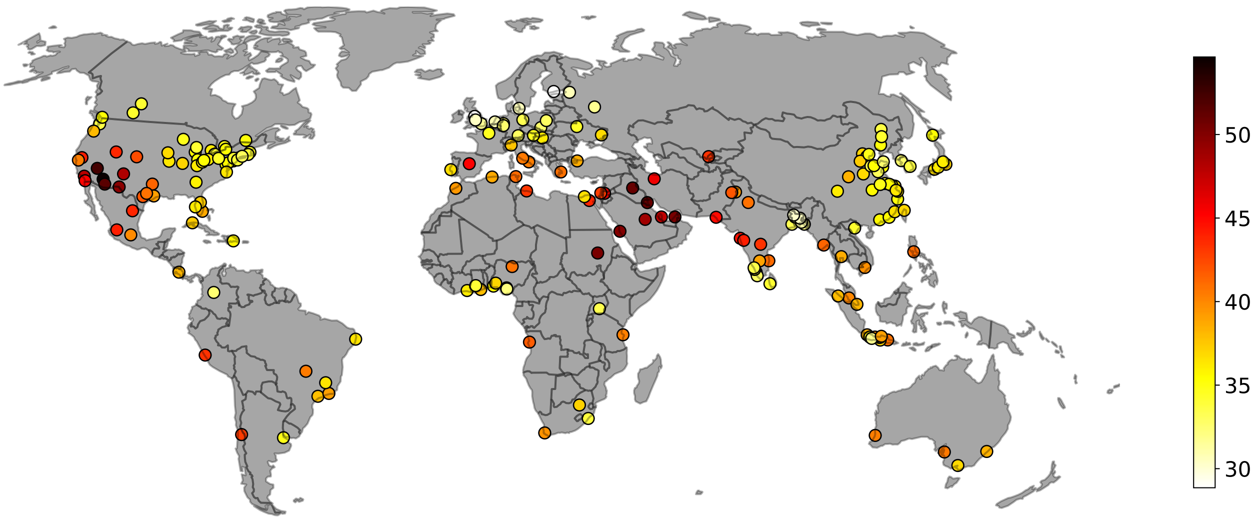

(a) Land Surface Temperature (LST) Daily thresholds values: LST_{90}^{d}

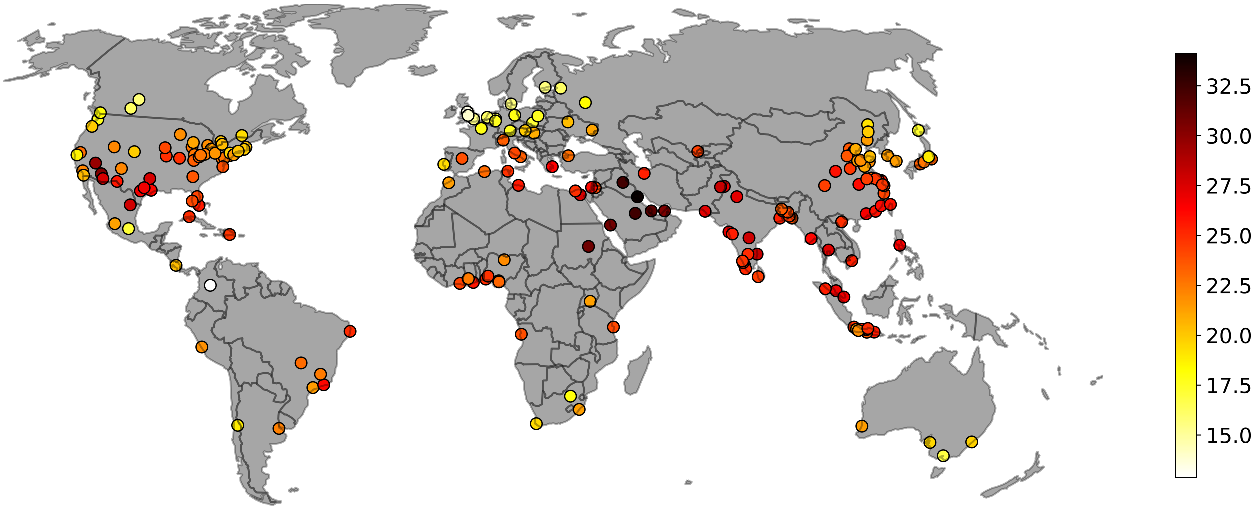

(b) Land Surface Temperature (LST) Night thresholds values: LST_{90}^{n}

Figure S3. Values of the Land Surface Temperature (LST) 90th+ percentile for daytime (a) and nighttime (b) across various cities based on their temporal distribution.

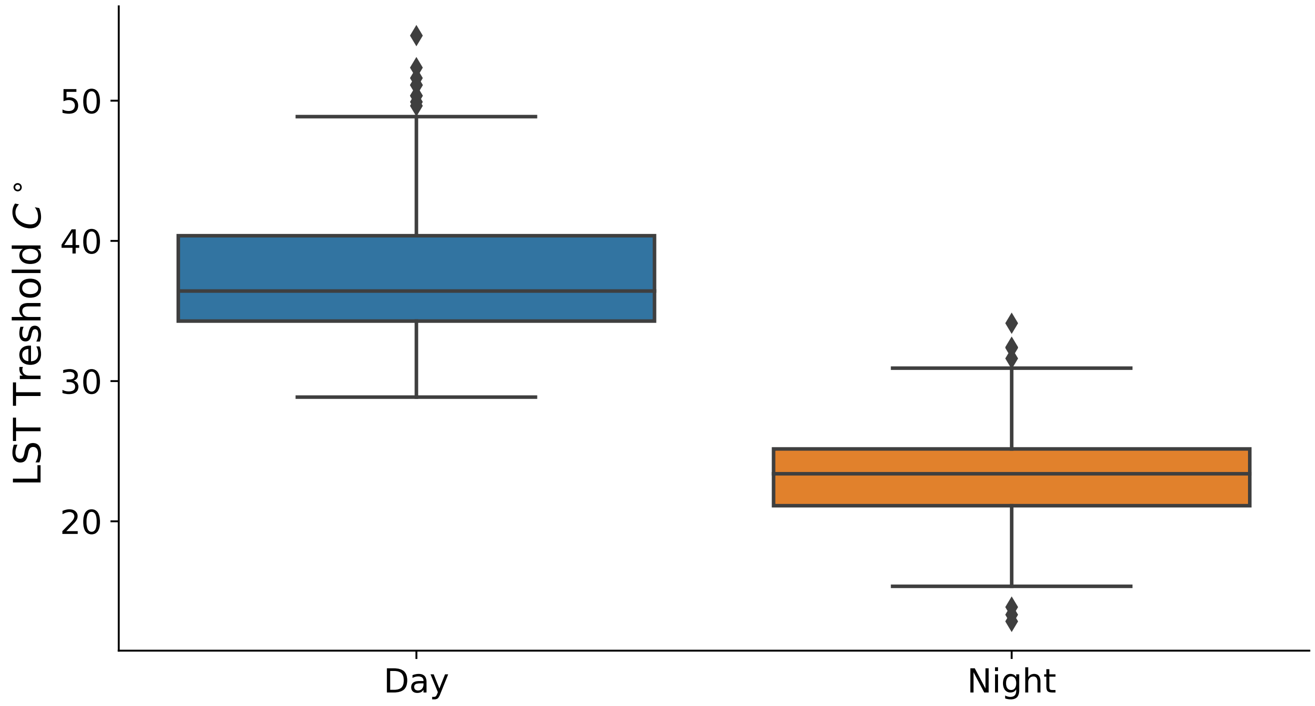

Figure S4. The boxplots illustrate the distribution of the daytime (Day) and nighttime (Night) values of the Land Surface Temperature (LST) thresholds of all cities.

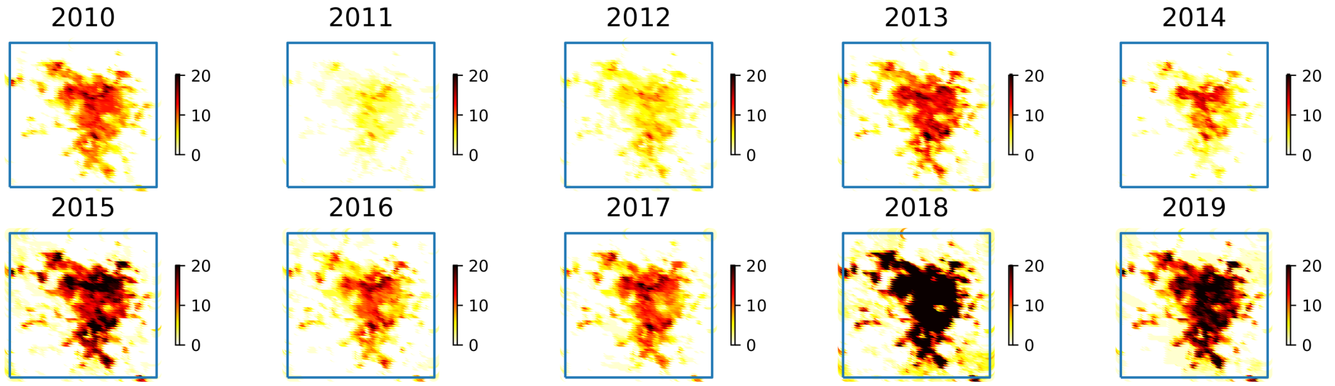

(a) Number of days in which over the daily value of the Land Surface Temperature (LST) is above the 90th percentile (LST^{d}_{90}).

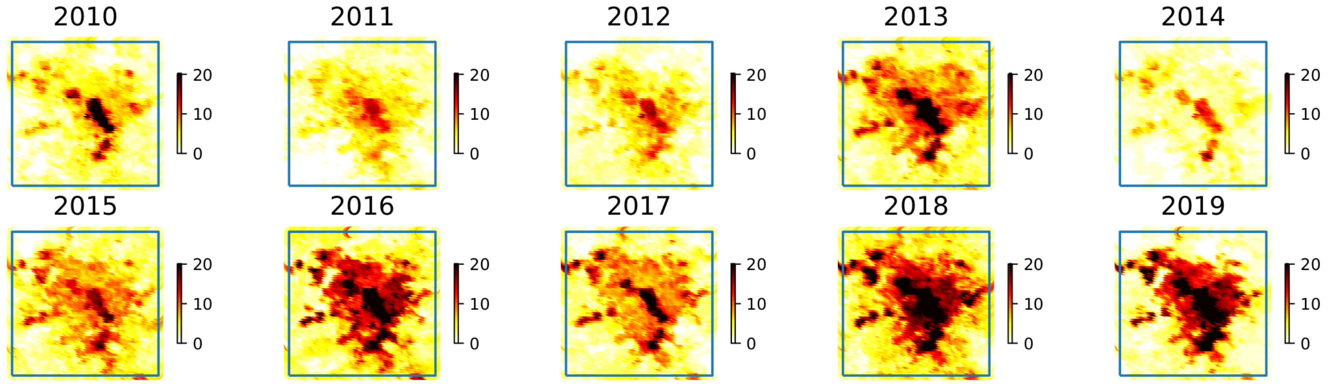

(b) Number of nights in which over the nighttime value of the Land Surface Temperature (LST) is above the 90th percentile (LST^{n}_{90}).

Figure S5. Number of days over the critical thresholds for the city of Paris.

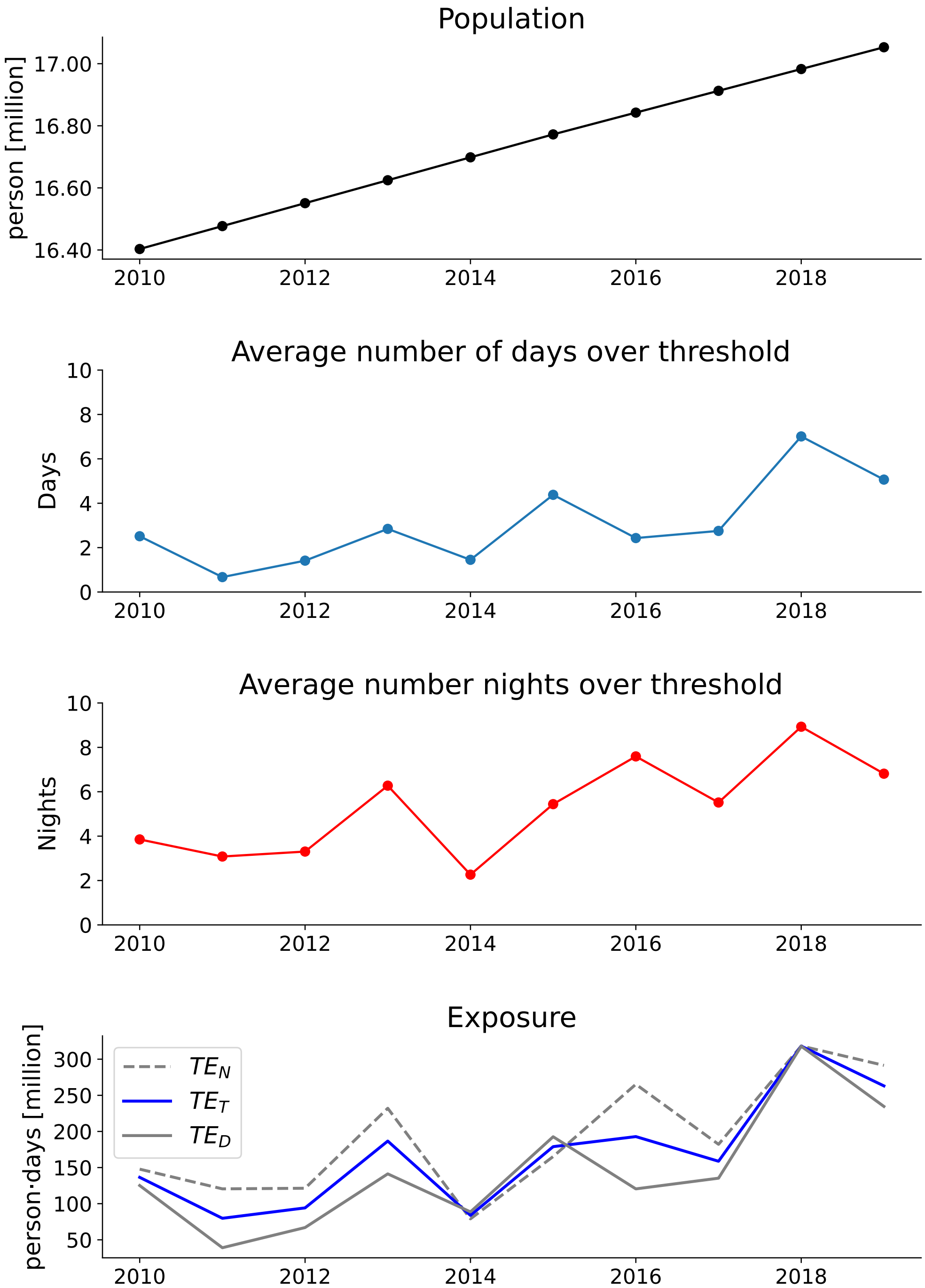

Figure S6. Definition of total exposure to extreme heat for the city of Paris over the different years. From top to bottom: population, average number of days over threshold, average number of nights over threshold and exposures.

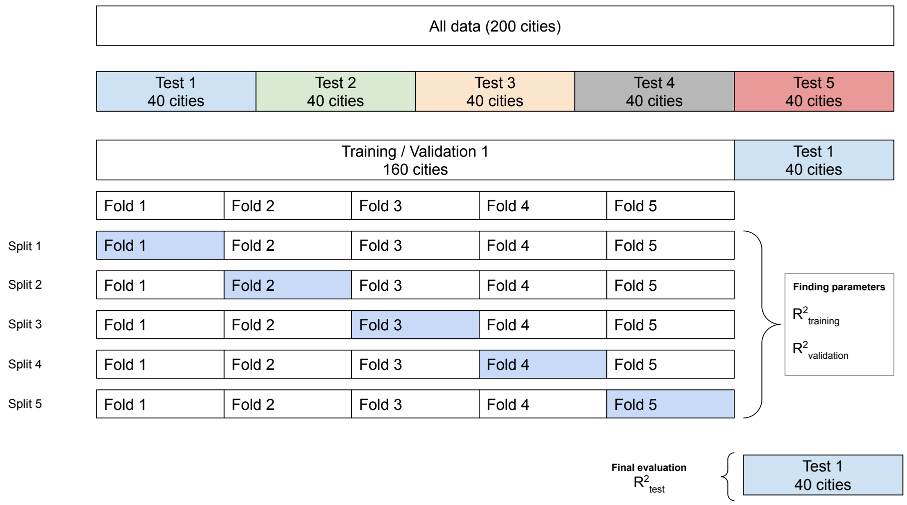

Figure S7. Schematic representation of the cross validation setting.

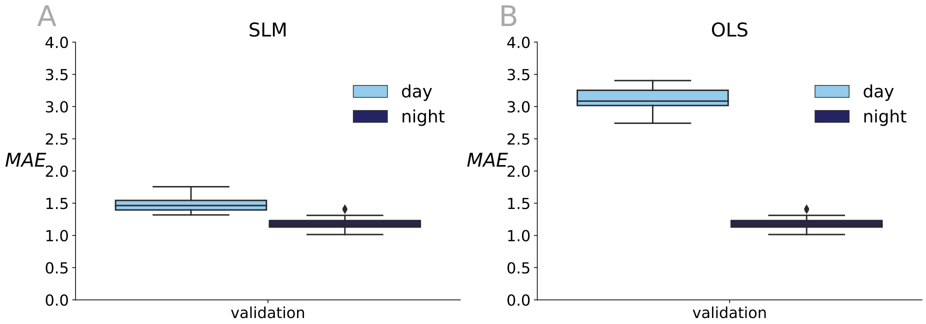

Figure S8. Mean Absolute Error (MAE) for the A) Spatial Lag Model (SLM) and B) Ordinary Least Squares (OLS) models.

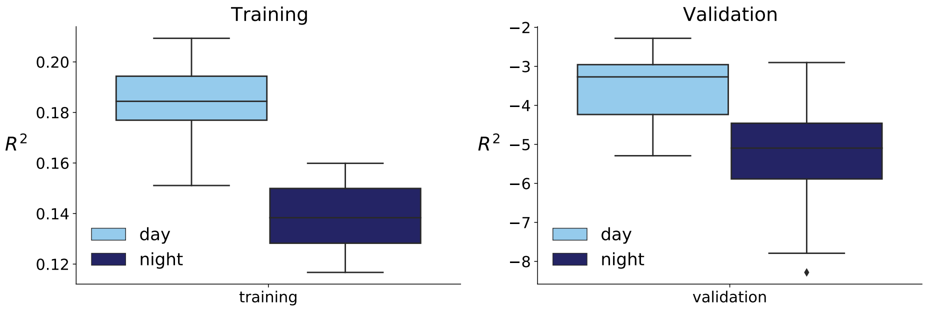

Figure S9. Values of the R^2 during the training and validation phase for an Ordinary Least Squares (OLS) regression model.

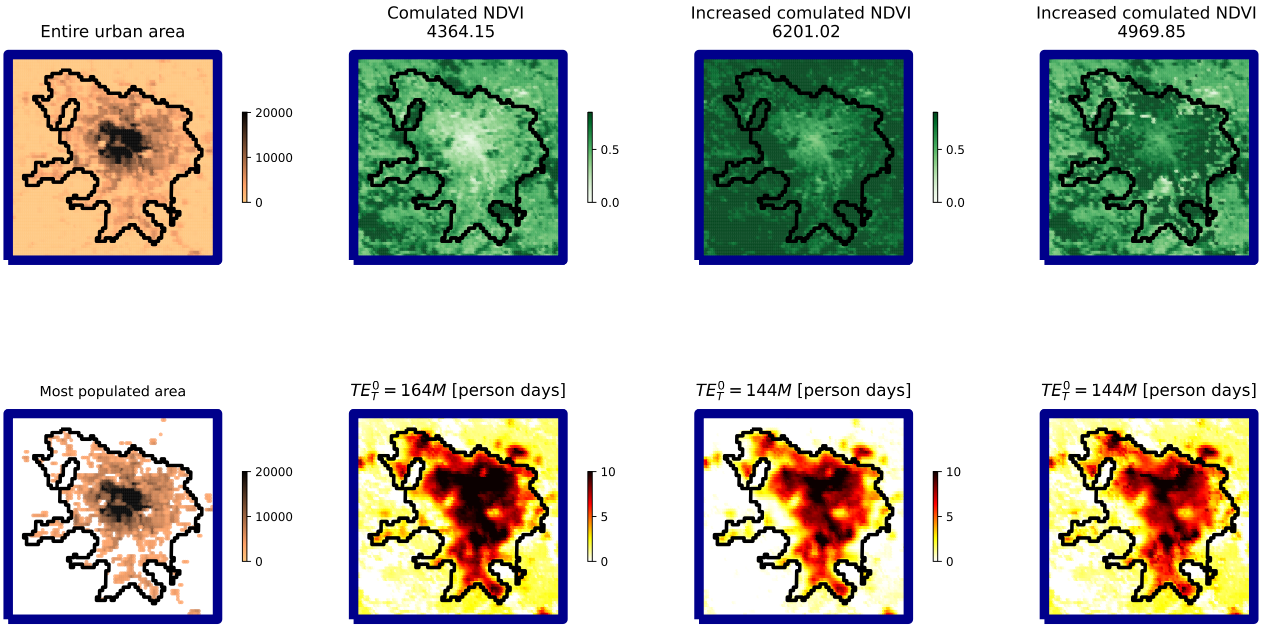

Figure S10. Values of Normalized Difference Vegetation Index (NDVI) and total exposure for different scenarios.

Supplementary Tables

Table S1. Diagnostics for spatial dependence: the mi/df ratio in PySAL [6] diagnostics for spatial dependence is the ratio of the Moran's I statistic to its degrees of freedom. This ratio is used to determine whether the spatial dependence in the data is statistically significant. A ratio greater than 1 indicates positive spatial autocorrelation, and a ratio less than 1 indicates negative spatial autocorrelation. If the ratio is close to 1, it suggests that there is no significant spatial autocorrelation in the data.

Test	Value	MI/DF	Prob
Moran's I	1482.912	0.93	0.0000
Lagrange Multiplier (lag)	2189960.088	1	0.0000
Robust LM (lag)	2543.394	1	0.0000
Lagrange Multiplier (error)	2198994.929	1	0.0000
Robust LM (error)	11578.235	1	0.0000
Lagrange Multiplier (SARMA)	2201538.323	2	0.0000

Table S2. Exposure reduction values for the city of Paris

Case	Region	Cumulated NDVI	Local NDVI increment	Total NDVI increment [%]	Total exposure TE_T [million person·days]	ΔTE_T [%]
Observed	Entire city	4364.15	0	0	164	0
Scenario 1	Entire city	6201.02	0.3	42	144	-12
Scenario 2	Most populated	4969.85	0.38	14	144	-12

Supplementary References

1. Beck, H. E. et al. Present and future köppen-geiger climate classification maps at 1-km resolution. Sci. data 5, 1–12379 (2018).

2. Anselin, L. & Rey, S. J. Spatial econometrics in an age of CyberGIScience. Int. J. Geogr. Inf. Sci. 26, 2211–2226 (2012).

3. Rey, S. J. & Anselin, L. Pysal: A python library of spatial analytical methods. In Handbook of applied spatial analysis:413 Software tools, methods and applications, 175–193 (Springer, 2009).

4. Anselin, L. & Bera, A. K. Spatial dependence in linear regression models with an introduction to spatial econometrics.411 Stat. textbooks monographs 155, 237–290 (1998).

5. Pesaresi, M. et al. Operating procedure for the production of the global human settlement layer from landsat data of the epochs 1975, 1990, 2000, and 2014. Publ. Off. Eur. Union 1–62 (2016).

6. Rey, Sergio J., and Luc Anselin. "PySAL: A Python library of spatial analytical methods." In Handbook of applied spatial analysis: Software tools, methods and applications, pp. 175-193. Berlin, Heidelberg: Springer Berlin Heidelberg (2009).
